# Global surgery, obstetric, and anaesthesia indicator definitions and reporting: An Utstein consensus report

**DOI:** 10.1371/journal.pmed.1003749

**Published:** 2021-08-20

**Authors:** Justine I. Davies, Adrian W. Gelb, Julian Gore-Booth, Janet Martin, Jannicke Mellin-Olsen, Christina Åkerman, Emmanuel A. Ameh, Bruce M. Biccard, Geir Sverre Braut, Kathryn M. Chu, Miliard Derbew, Hege Langli Ersdal, Jose Miguel Guzman, Lars Hagander, Carolina Haylock-Loor, Hampus Holmer, Walter Johnson, Sabrina Juran, Nicolas J. Kassebaum, Tore Laerdal, Andrew J. M. Leather, Michael S. Lipnick, David Ljungman, Emmanuel M. Makasa, John G. Meara, Mark W. Newton, Doris Østergaard, Teri Reynolds, Lauri J. Romanzi, Vatshalan Santhirapala, Mark G. Shrime, Kjetil Søreide, Margit Steinholt, Emi Suzuki, John E. Varallo, Gerard H. A. Visser, David Watters, Thomas G. Weiser

**Affiliations:** 1 Institute of Applied Health Research, University of Birmingham, Birmingham, United Kingdom; 2 Centre for Global Surgery, Department of Global Health, Stellenbosch University, Stellenbosch, South Africa; 3 Department of Public Health, Wits University, Johannesburg, South Africa; 4 World Federation of Societies of Anaesthesiologists, London, United Kingdom; 5 Department of Anesthesia & Perioperative Care, University of California San Francisco, California, United States of America; 6 Department of Anesthesia & Perioperative Medicine, Western University, London, Ontario, Canada; 7 Department of Anaesthesia and Intensive Care Medicine, Baerum Hospital, Sandvika, Norway; 8 Dell Medical School, University of Texas at Austin, Austin, Texas, United States of America; 9 Institute for Strategy and Competitiveness, Harvard Business School, Boston, Massachusetts, United States of America; 10 Division of Paediatric Surgery, The National Hospital, Abuja, Nigeria; 11 National Surgical, Obstetric and Anaesthesia Planning Committee, Federal Ministry of Health, Abuja, Nigeria; 12 Department of Anaesthesia and Perioperative Medicine, Groote Schuur Hospital, Cape Town, South Africa; 13 Department of Anaesthesia and Perioperative Medicine, University of Cape Town, Western Cape, South Africa; 14 Research Department of Community Medicine, Stavanger University Hospital, Stavanger, Norway; 15 School of Medicine, College of Health Sciences, Addis Ababa University, Ethiopia; 16 Faculty of Health Sciences, University of Stavanger, Stavanger, Norway; 17 Critical Care and Anaesthesiology Research Group, Stavanger University Hospital, Norway; 18 NoBrainerData, Maryland, United States of America; 19 Paediatric Surgery, Department of Clinical Sciences in Lund, Faculty of Medicine, Lund University, Lund, Sweden; 20 Department of Anesthesia, Intensive Care Medicine, Interventional Pain Unit, Hospital Del Valle, San Pedro Sula, Honduras; 21 Department of Global Public Health, Karolinska Institutet, Stockholm, Sweden; 22 Department of Neurosurgery, Loma Linda University, Loma Linda, California, United States of America; 23 Population and Development, United Nations Population Fund, New York, New York, United States of America; 24 Program in Global Surgery and Social Change, Harvard Medical School, Boston, Massachusetts, United States of America; 25 Anesthesiology and Pain Medicine, Health Metrics Sciences, Global Health, and Institute for Health Metrics and Evaluation, University of Washington, Seattle, Washington, United States of America; 26 Laerdal Foundation, Stavanger, Norway; 27 King’s Centre for Global Health and Health Partnerships, School of Population Health and Environmental Sciences, King’s College London, London, United Kingdom; 28 Center for Health Equity in Surgery and Anesthesia, University of California San Francisco, San Francisco, United States of America; 29 Department of Surgery, Institute of Clinical Sciences, Sahlgrenska Academy, University of Gothenburg, Gothenburg, Sweden; 30 SADC-Wits Regional Collaboration Centre for Surgical Healthcare (WitSSurg), Department of Surgery, University of the Witwatersrand, Johannesburg, South Africa; 31 Department of Plastic and Oral Surgery, Boston Children’s Hospital, Boston, Massachusetts, United States of America; 32 Department of Anesthesiology and Pediatrics, Vanderbilt University Medical Center, Tennessee, United States of America; 33 AIC Kijabe Hospital, Kenya; 34 Copenhagen Academy for Medical Education and Simulation, The University of Copenhagen, Copenhagen, Denmark; 35 Clinical Services and Systems, Integrated Health Services, World Health Organization, Geneva, Switzerland; 36 Department of Anaesthesia and Perioperative Care, Guy’s and St. Thomas’ Hospital, London, United Kingdom; 37 Institute of Global Surgery, Royal College of Surgeons in Ireland, Dublin, Ireland; 38 Department of Gastrointestinal Surgery, Stavanger University Hospital, Stavanger, Norway; 39 Department of Clinical Medicine, University of Bergen, Norway; 40 Helgeland Hospital Trust, Sandnessjøen, Norway; 41 Norwegian University of Science and Technology, Trondheim, Norway; 42 The World Bank, Washington, DC, United States of America; 43 Department of Safe Surgery, Jhpiego, Baltimore, Maryland, United States of America; 44 Department of Obstetrics, University Medical Center, Utrecht, the Netherlands; 45 University Hospital Geelong, Victoria, Australia; 46 Faculty of Health, School of Medicine, Deakin University, Victoria, Australia; 47 Royal Australasian College of Surgeons, Melbourne, Victoria, Australia; 48 Stanford University School of Medicine, Department of Surgery Division of General Surgery, Section of Trauma & Critical Care Stanford University, Stanford, United States of America; 49 Department of Clinical Surgery, University of Edinburgh, Edinburgh, Scotland

## Abstract

**Background:**

Indicators to evaluate progress towards timely access to safe surgical, anaesthesia, and obstetric (SAO) care were proposed in 2015 by the Lancet Commission on Global Surgery. These aimed to capture access to surgery, surgical workforce, surgical volume, perioperative mortality rate, and catastrophic and impoverishing financial consequences of surgery. Despite being rapidly taken up by practitioners, data points from which to derive the indicators were not defined, limiting comparability across time or settings. We convened global experts to evaluate and explicitly define—for the first time—the indicators to improve comparability and support achievement of 2030 goals to improve access to safe affordable surgical and anaesthesia care globally.

**Methods and findings:**

The Utstein process for developing and reporting guidelines through a consensus building process was followed. In-person discussions at a 2-day meeting were followed by an iterative process conducted by email and virtual group meetings until consensus was reached. The meeting was held between June 16 to 18, 2019; discussions continued until August 2020. Participants consisted of experts in surgery, anaesthesia, and obstetric care, data science, and health indicators from high-, middle-, and low-income countries. Considering each of the 6 indicators in turn, we refined overarching descriptions and agreed upon data points needed for construction of each indicator at current time (basic data points), and as each evolves over 2 to 5 (intermediate) and >5 year (full) time frames. We removed one of the original 6 indicators (one of 2 financial risk protection indicators was eliminated) and refined descriptions and defined data points required to construct the 5 remaining indicators: geospatial access, workforce, surgical volume, perioperative mortality, and catastrophic expenditure.

A strength of the process was the number of people from global institutes and multilateral agencies involved in the collection and reporting of global health metrics; a limitation was the limited number of participants from low- or middle-income countries—who only made up 21% of the total attendees.

**Conclusions:**

To track global progress towards timely access to quality SAO care, these indicators—at the basic level—should be implemented universally as soon as possible. Intermediate and full indicator sets should be achieved by all countries over time. Meanwhile, these evolutions can assist in the short term in developing national surgical plans and collecting more detailed data for research studies.

## Abstract 2

Justine Davies and colleagues discuss the development of updated indicators to support global achievement of access to safe surgical, anaesthetic, and obstetric care.

## Background

In 2015, The Lancet Commission on Global Surgery (LCoGS), Disease Control Priorities-3 Surgery, and World Health Assembly Resolution 68/15 on “Strengthening Emergency and Essential Surgical Care and Anaesthesia as a Component of Universal Health Coverage” showed the dire global state of surgical and anaesthesia care provision globally and the necessity for large and rapid improvements, especially in many low- or middle-income countries (LMICs) [1,2].

Given that there were no widely accepted indicators used to track progress towards improved timely access to quality surgical and anaesthetic care, members of LCoGS proposed a set of 6 indicators (Table A in S1 File) for this purpose. These were to be used as a set to illustrate access and quality and broadly classified under preparedness for care (access to timely surgery and workforce density), delivery of surgical and anaesthesia care (surgical volume and perioperative mortality), and effect of surgery and anaesthesia (protection against catastrophic expenditure and protection against impoverishing expenditure). These indicators were rapidly adopted into the WHO’s 100 Basic Global Health Indicators and the World Bank’s World Development Indicators [3,4]. They have also been used in research studies to assess the state of provision of surgical care in multiple country settings and proposed for use by ministries of health to assess progress towards improving surgical care nationally [5–10].

However, although widely accepted as valuable indicators, the LCoGS only broadly defined them, leaving much flexibility in the choice of data points from which to derive them [11,12]. Given that each indicator is formed from multiple data points (for example, perioperative mortality requires assessment of death, the time of death, and, potentially, the risk of death for patients undergoing surgery), lack of clarity has resulted in confusion and delays in data collection, and difficulty in comparing results among countries and over time [11]. Indeed, recently, an assessment of country-level indicator reporting found poor availability and heterogeneous definitions, which limited comparability and utility of the indicators. When using the indicators put forward by LCoGS, although 154 countries out of the WHO member states had data on workforce, only 19 had data on timely access to a facility capable of providing surgical care, 72 had data on the numbers of procedures done, and 9 had data on perioperative mortality. No country had empirical data on the 2 indicators of financial risk from surgery and anaesthesia. Even for the most available indicator of workforce, definitional issues limited its comparability across countries and its utility [11]. For perioperative mortality, there were several different reporting times in use, i.e., 24-hour mortality, 7-day mortality, in-hospital mortality, 30-day mortality, or surgical mortality. Although some studies have been done to collect LCoGS indicators in a few settings since LCoGS was published, these have typically been done as one-off research projects with the numbers of personnel and funding available to researchers which may not be available to local governments. Additionally, the lack of definitional clarity in the original LCoGS indicators has meant that researchers have developed their own definitions of some indicators, making results difficult to harmonise for use in cross-country modelling studies or meta-analyses [8,10,11–13]. This greatly hinders the ability to produce or assess local, national, or international achievement of global targets for surgery.

Our aim was therefore to bring experts in surgical, obstetric, and anaesthesia clinical care and academia together with global indicators experts, data scientists, and policy makers to appraise the existing indicators; refine their descriptions; and define data points needed for their derivation. The intention was to both reinforce and clarify the global indicator set to facilitate their use in research, national planning, and global health development and advocacy, the latter in order to improve political priority for global surgery.

## Methodology

We assembled an international group of experts in policy; surgery, anaesthesia, obstetrics; and data science for an in-person meeting to develop consensus using the principles of the Utstein process [14–18]. Previous Utstein initiatives have focused on defining core outcome sets for out-of-hospital cardiac arrest and cardiopulmonary resuscitation, time points at which they should be collected, and the way in which they should be reported. Our aim was to bring this Utstein evidence-based rigour and consensus-informed consolidation to global surgery indicators.

The meeting took place at the Utstein Abbey, Mosteroy Island, Norway, on June 16 to 18, 2019. The meeting was followed up by email correspondence among all members of the panel and virtual group discussions to resolve ongoing issues. Discussions continued from the end of the meeting up until August 2020.

### Panel selection

The steering committee (Table B in S1 File) identified potential participants with relevant experience to help define surgery and anaesthesia metrics, and/or using global health metrics in practice, and considering country income strata. Snowball sampling was used to identify further participants with expertise. A total of 60 potential participants were identified; however, meeting places were limited to 40. Therefore, participants were shortlisted by the steering committee based upon the relevance of their expertise; if any from the first 40 could not attend, they were either asked to nominate a suitable substitute, or the next on the list was invited.

### Preparation

Prior to the meeting, relevant literature on global surgery/anaesthesia and indicators were sent to participants [1,7,8,10,11–13,19–27]. In addition, all participants were sent information on guiding principles previously used to establish global surgery indicators (Table 1) [22]. Members were informed that the purpose of the meeting was to appraise, revise, and define, but not necessarily abandon, the existing indicators that have already garnered global momentum. Members were also encouraged to share their own experience of indicator collection in their own specialty fields and recommendations, successes, and failures informed development of these indicators.

**Table 1:** Guiding principles for global surgery indicators [22].

**Simplicity** Indicators should be simple, clear, and inexpensive to obtain from hospitals, providers, professional societies, and governmental agencies. Health resources should not be diverted or unduly burdened by demands for data collection.
**Wide applicability** Indicators should use definitions relevant to the span of surgical care worldwide. They should also be meaningful to health professionals, researchers, and policy makers and provide information allowing reasonable conclusions on the state of surgical services within a country.
**Relevance to public health** Surgical indicators should incorporate measures of access and outcome. They should provide indicators likely to respond to substantial changes in the delivery or quality of surgical care.
**Unintended negative consequences of measurement reduced to a minimum** Potentially negative consequences should be considered, since scrutiny can result in perverse effects, driving practice patterns that bolster statistics at the expense of patient care.

### Consensus process

Methods were in accordance with Utstein methodology on developing reporting guidelines [14–17] and other guidelines for developing reporting criteria [18]. Utstein-style conferences use an established process to consolidate definitions and reporting criteria to improve comparability of outcomes reported in studies, databases, demographic surveys, and administrative reports. The resulting outputs are guidelines and templates that can be adopted by governments, policy makers, journals, demographers, and researchers as unifying reporting criteria. This ensures global consistency and comparability across data types, definitions, and reporting style.

The steering committee assigned attendees to one of 6 working groups based on knowledge and expertise. Each working group related to one indicator; access, volume, workforce, and perioperative mortality rate (POMR). Catastrophic and impoverishing expenditure was discussed by one group, given their similarity. The sixth group, entitled the “Parking Lot,” was included to address gaps in the current set of indicators that should be further developed in future iterations of the Utstein consensus and/or through future research—results of this group are not presented here but will be used for future evolutions of the indicators.

Each group was assigned a lead and a deputy based on their leadership in the indicator under consideration (Table B in S1 File). The group lead presented an outline of the current definition of the indicator [1] and issues found in its availability, comparability, and utility [11]. After which, the groups were asked to develop a clear overall definition for each indicator and consider the overarching data points needed to derive it. Then, given the potential levels of granularity and complexity inherent in each indicator, each group was asked to consider minimum basic data points (basic) to allow global comparisons using nationally led data collection initiatives. To be defined as basic, we agreed that reporting at the country level should be feasible within the next 2 years. We then asked groups to consider how these data points should evolve to intermediate (2 to 5 years) and full (>5 years) sets, which can be used to guide collection of harmonisable in-depth data that are feasible in well-resourced settings or research studies and that will aid policy making at the national level. The evolutionary time frame was agreed as a guideline for countries that are not yet able to collect the evolved data sets and should not be seen as prohibiting countries that are already able to produce these data sets from collecting them.

Each indicator working group was initially divided in half to address the indicator independently and then reconvened as a complete group to compare notes and recommendations. After agreement within the working group, each presented their suggestions to the full panel in a plenary session to build consensus across all attendees. Thus, all participants contributed to the discussions on each of the indicators. Key points of discussion and the outcomes of the plenary discussions were recorded by the working group lead and deputy.

After the meeting, each working group lead and deputy entered the discussion results for their indicator into a template. Templates were compiled by the writing group and then circulated to all attendees for feedback. Comments were again compiled by the writing group who, on discussion, further refined indicators or their data points to ensure that there was consistency in reporting across all indicators. After this process, any adjustments were sent to all attendees for further feedback and then correction by the writing group, until consensus was built. Where disagreement remained after this process, small working groups of panel members with relevant expertise and those who were in disagreement were assembled to enable consensus to be reached, facilitated by a writing group member. Once agreement had been reached in these discussions, the conclusion of the discussion and any adjustments suggested were sent to all participants by email to assess their agreement with the proposal.

## Results

Out of 60 participants invited, 24 declined due to other commitments. After substitutes were nominated for some participants, 38 participants attended the meeting; country of origin and specialty are shown in Table 2. Working group members and leads are shown in Table B in S1 File. Small group discussions to resolve disagreement around POMR, workforce definitions, the inclusion of Bellwether procedures, and the time frame for the evolution of the indicators (Text A in S1 File) were complete by August 2020, after which, no disagreements remained.

**Table 2:** Country of origin and specialty of meeting attendees. Note that 2 of the attendees work in multiple settings, EM in Zambia and South Africa, and JD in the UK and South Africa; hence, numbers do not add up to 38.

Role	Number
Surgery	14
Anaesthesia	12
Demography, Statistics, and Policy [WHO; United Nations Population Fund; World Bank; United States Agency for International Development, Demographic and Health Surveys; United Nations Statistical Commission]	5
Obstetrics	3
Global Health Expert	3
General physician	1
**Country of residence of attendees**	
Country	Number
Australia	1
Canada	1
Denmark	1
Ethiopia	1
Germany	1
Honduras	1
Nigeria	1
Norway	6
South Africa	4
Sweden	3
the Netherlands	1
UK	3
USA	15
Zambia	1

There was consensus that the overarching descriptions and data points used to derive all indicators required further clarification in order to improve their availability, comparability, and utility for research, reporting at national or international level, and national and international planning. The meeting resulted in changing the overarching descriptions of the indicators, and, importantly, the panel reached consensus on data points and how to use these to derive all indicators across 3 progressive levels: basic, intermediate, and full, whereas these data points were previously not defined.

Table 3 shows, for each indicator, the original LCoGS overarching description, the Utstein revised description, a summary description of the data points required to construct the indicator, and the basic (<2 year) data points needed to construct the indicator. Tables C-G in S1 File contain these parameters for intermediate and full data sets.

**Table 3:** Revised LCoGS indicators and basic data points.

**Indicator 1: Geospatial access**	
**LCoGS indicator definition**	Proportion of the population that can access, within 2 hours, a facility that can do cesarean delivery, laparotomy, and treatment of open fracture (the Bellwether procedures)
**Utstein revised definition**	**Proportion of a country’s population with geographic access (within 2 hours) to a facility capable of providing surgical and anaesthesia care for the Bellwether procedures (cesarean section, laparotomy, and surgical management of open long bone fracture)**
**Overall summary of data elements**	• **Population estimates** • **Facility locations** • **Capacity of health facilities to do Bellwether procedures** • **Distance and travel time of population to facilities**
**Basic data points needed to construct the indicator (<2 years)**	Population - Population data or modelled estimates at resolution of 1 × 1 km (disaggregated by 5-year age groupings and sex, if available) Facility location/capability - Location of health facilities offering Bellwether procedures Distance/travel time^3.1^ - Estimated time to travel to facilities from population locations
**Indicator 2: Workforce**	
**LCoGS indicator definition**	Number of specialist surgical, obstetric, and anaesthetic physicians who are working per 100 000 population.
**Utstein revised definition**	**Number of each of surgery, obstetric, or anaesthesia providers who are actively practicing, per 100 000 population**
**Overall summary of data elements**	• **Provider**^**3.2**^ **numbers as:** - Number of *nationally certified*^*3*.*3*^ *specialist physician*^*3*.*4*^ *practitioners* for each of surgery, obstetric, or anaesthesia care, excluding trainees - Number of *nationally* c*ertified nonspecialist physician practitioners* of surgery, obstetric, or anaesthesia care, excluding trainees - Number of *nationally certified nonphysician practitioners* of surgery, obstetric, or anaesthesia care, excluding trainees - Number of *other practitioners (“other practitioners”)* of surgery, obstetric, or anaesthesia care who do not fit into aforementioned categories (includes physician trainees and noncertified nonphysician providers—note this is not used in the basic data) • **Total country population**
**Basic data points needed to construct the indicator (<2 years)**	Providers - Total number of nationally certified specialist physician practitioners for each of surgery, anaesthesia, or obstetric care Disaggregated by cadre (surgery, obstetric, or anaesthesia providers) - Total number of other nationally certified providers of surgery, obstetric, or anaesthesia care Disaggregated by cadre (surgery, obstetric, or anaesthesia providers) Population - Total country population
**Indicator 3: Volume**	
**LCoGS indicator definition**	Number of procedures done in an operating theatre, per 100 000 population per year
**Utstein revised definition**	**Number of surgical procedures done in an operating theatre using any form of anaesthesia** ^**3.5**^ **, per 100,000 population per year**
**Overall summary of data elements**	• **Number of surgical procedures done in an operating theatre, using any anaesthesia, per year** • **Total country population**
**Basic data points needed to construct the indicator (<2 years)**	Procedures - Total number of procedures done in an operating theatre using any form of anaesthesia, per year^3.5^ Population - Total country population
**Indicator 4: POMR**	
**LCoGS indicator definition**	All-cause death rate before discharge in patients who have undergone a procedure in an operating theatre using any form of anaesthesia, divided by the total number of procedures, presented as a percentage, per year
**Utstein revised definition**	**Deaths from all causes, before discharge (up to 30 days), in all patients who have received any anaesthesia for a procedure done in an operating theatre** ^**3.5**^ **, divided by the total number of procedures, per year, expressed as a percentage**
**Overall summary of data elements**	• **Number of patients undergoing a surgical procedure in an operating theatre using any form of anaesthesia who died before hospital discharge (up to 30 days), per year** • **Number of procedures done in an operating theatre, using any anaesthesia, per year (from Indicator 3: Volume)**
**Basic data points needed to construct the indicator (<2 years)**	Deaths - Number of in-hospital deaths (up to 30 days) in all patients who received any anaesthesia for a surgical procedure performed in an operating theatre^3.5^, per year Procedures - Number of surgical procedures done in an operating theatre using any form of anaesthesia, per year^3.5^ Time point: - Deaths before discharge (up to 30 days)
**Indicator 5: FRP**	
**LCoGS indicator definition**	FRP: Risk of Catastrophic Expenditure from Surgical Care
**Utstein revised definition**	**Percentage of the population at risk of catastrophic expenditure *if* they were to require a surgical procedure** ^**3.6**^
**Overall summary of data elements**	• ***OOP***^**3.7**^ OOP is the *direct medical* costs incurred from receiving surgical care from time of admission to a facility capable of providing surgical and anaesthesia care to the time of discharge. • **Household expenditure** Total household expenditure (Y) is defined as “the sum of the monetary values of all items (goods and services) consumed by each household” over 12 months. **Catastrophic expenditure threshold** • The catastrophic expenditure threshold should be set at 10% of total household expenditure^3.8^. ***If (OOP/Y) × 100 >10*, *catastrophic expenditure has occurred***
**Basic data points needed to construct the indicator (<2 years)**	OOP expenditure for access to surgical care - Nationally representative survey of direct OOP expenditure Household expenditure - National total household expenditure (per individual household)

The basic data sets are for use for global reporting at the macrolevel only since they provide insufficient granularity to inform national planning or service refinement at the meso- or microlevel. For example, the basic data set does not provide meaningful comparison of POMR across settings since the results are not adjusted for baseline patient risk or type of procedure.

^3.1^For comparability, travel time means ideal time to travel between a location and a facility. It does not mean experienced travel time from recognition of the need for surgery to arriving at a facility, which may incorporate delays in seeking care or delays in obtaining transport.

^3.2^We have not provided a definition of what a surgery, anaesthetic, or obstetric provider is; we agreed that these should be defined by each country, with recognition that the definitions are likely to vary locally. Providers are persons directly involved in delivering the surgery, obstetric, or anaesthetic care, i.e., the person doing the operation or giving the anaesthetic.

^3.3^Certified means completion of a government and/or professionally approved advanced education program that leads to a nationally recognised qualification to provide surgery, anaesthesia, or obstetric care.

^3.4^Specialist physicians are providers who have obtained a medical degree (physician) and undergone specialty postgraduate training (certification).

^3.5^This recognises that, at the current time, definitions of procedures that constitute surgery differ between countries and data sources. We have therefore agreed upon a broad definition of procedures for the basic data set (<2-year time frame), without defining a list. This definition includes incision, excision, or manipulation of tissue needing anaesthesia in an operating theatre. This includes day-cases but excludes procedures in other locations, i.e., outside of the operating theatre. Definition of anaesthesia is regional or general anaesthesia, or profound sedation to control pain. Number of surgical codes in a single anaesthesia procedure are counted as one case. If only a subset of procedures is feasible to collect for this indicator, then the type of procedures included should be transparently reported.

^3.6^Catastrophic expenditure is usually calculated at the individual level (with data collected on OOP and household expenditure for each individual undergoing a medical admission episode). However, many people do not access surgery care because of fear of catastrophic expenditure. This indicator thus uses individual OOP expenditure for those who seek surgery in combination with national average level household expenditure to estimate the proportion of people who would suffer catastrophic expenditure *if* they were to need surgery.

^3.7^Direct OOP costs could, in reality, include prehospital direct medical costs. However, they are not included here as they are small relative to the hospitalisation episode and patients may not recall these as readily as hospitalisation costs. This does not include direct nonmedical costs (lodging, food, transport to and from facility). This does not include indirect costs (e.g., loss of earnings).

^3.8^We note as per SDG Target 3.8.2, there are 2 recognised thresholds, >10% and > 25%; however, we have chosen 10%.

FRP, financial risk protection; LCoGS, The Lancet Commission on Global Surgery; OOP, out-of-pocket expenditure; POMR, perioperative mortality rate.

The 2 indicators on effect of care were condensed into one: Risk of catastrophic expenditure on requirement for surgery replaces protection against catastrophic expenditure and protection against impoverishing expenditure. Use of catastrophic expenditure aligns with the expenditure indicator used in the Sustainable Development Goals and is a key indicator to monitor progress towards universal health coverage.

Regarding changes to the indicator descriptions, the panel agreed that the original indicator access to timely essential surgery should be changed to geospatial access to a facility that has capacity to deliver surgery and anaesthesia care for Bellwether procedures. This is in order to reduce the potential dimensions inherent in the broad concept of access—for example, cultural, quality, and financial—noting that clinical quality and financial dimensions are covered by other indicators. We agreed that the data points for constructing this indicator at the basic level should allow estimation of the proportion of the population who would have geospatial access to a facility were they to need care. While realised access (a person who needs care actually accesses it) may be feasible to measure in some countries, given the complexity of collecting this data in countries with underdeveloped health systems, the consensus was that this should not form part of the basic data set, but data points needed to consider access in more granularity are included in the full data set. Information on whether a facility provides the Bellwether procedures (originally cesarean section, laparotomy, and treatment of an open fracture) is a necessary component of this indicator. The Bellwethers were developed as a marker of a hospital, which, if all 3 were provided, could deliver a broad base of surgical care [1]. Although these procedures have been collected as part of research studies, we agreed that their utility for national reporting was limited by lack of definitional clarity, especially for treatment of an open fracture. We discussed this issue at length, including whether we should remove the concept of Bellwethers from this indicator, however, ultimately reached consensus that they should be included, with clarity that treatment of an open fracture should become surgical management of an open long bone fracture. The group also considered the use of a basket of surgical procedures, akin to the consumer price index concept where a core “basket” of goods is used across multiple countries to collect data on volume purchased and price of these goods. Some of the panellists were working on an international Delphi to define such a basket, but that was only published after this meeting [28]. It was also agreed that to inform the indicators, such work would require further acceptance by the global community and further consensus.

The main consensus change to the specialist surgical workforce density indicator was to include all cadres providing surgical, obstetric, and anaesthesia care in the definition, broadening this out from being limited to the physician workforce to now including other nationally certified (nonphysician practitioners). We also improved clarity in the definition of providers in order to allow evolution of granularity.

The panel agreed that the potential breadth of procedures that can be defined under the umbrella of surgery limits the comparability and utility of the surgical volume indicator [11,21]. For the basic data set, we have therefore defined surgical procedures in broad terms as procedures done in an operating theatre. These include incision, excision, or manipulation of tissue using anaesthesia in an operating theatre, including day-cases but excluding surgical procedures in other locations, i.e., outside of the operating room. Definition of anaesthesia is regional or general anaesthesia, or profound sedation to control pain during the procedure. We agreed upon a structure to increase the granularity of the data collected over time (Tables C-G in S1 File), acknowledging that while providers and operating theatres often capture detailed data on procedures done, in many LMIC facilities, these data are held in handwritten log books and are difficult to extract for monitoring purposes.

Regarding POMR—the only clinical indicator in the LCoGS indicators—we had disagreement about whether the indicator should simply be that countries are collecting information on POMR, given the potential adverse consequences of reporting high POMR (Text A in S1 File). However, after discussion, we reached consensus that there was utility in reporting POMR, although for global accountability processes, this should be at a national rather than hospital level. There was consensus that for the basic data set, the time period for reporting should be in-hospital, rather than 30-day mortality, which is a standard indicator reported in high-income countries. This is due to strong evidence that mortality out to 30 days is generally currently not available in many countries and that 70% to 80% of POMR occurs in-hospital and 20% to 30% after discharge [29,30]. We also agreed that risk adjustment is not currently possible for many countries that lack data related to procedure type and patient risk (derived using the American Society of Anaesthesia [ASA] score); therefore, at the basic level, POMR should not be risk adjusted. We noted that lack of risk adjustment will limit comparisons across countries given the presence of differences in risk between country populations. Comparisons may become feasible at the intermediate and full level, when we agree that covariates for risk adjustment at the patient level should also be collected.

Discussions around the indicator on catastrophic expenditure centred on the nearly universal lack of data points from which to derive this indicator, especially in LMICs. For example, documented hospital costs of procedures often grossly underrepresent the full extent of direct medical costs, patients may not be aware of their household expenditure, or people who are impoverished may not access surgery care at all. To rigorously collect these data requires doing exit interviews with patients. However, we recommend that at the least, data on costs of surgery are collected using nationally representative surveys where reliable information on costs of care are not available from other sources. We agreed to not include indirect costs of accessing care until later evolutions of the indicators, given the difficulty in collecting information on earnings (and their loss) especially in settings where many people work in the informal or subsistence sectors. To overcome difficulty in ascertaining an individual’s household expenses, we recommend the use of national household expenditure, which will allow estimation of the proportion of the population who would be at risk of catastrophic expenditure if they were to need surgery. We thus agreed to change the overarching description of this indicator to “Percentage of the population at risk of catastrophic expenditure if they were to require care for a surgical procedure.” We recognised that it doesn’t capture nonmedical direct and indirect costs, e.g., those incurred in accessing care, but the difficulty in collecting these means they are not feasible for the basic or intermediate data sets.

## Discussion

The meeting attendees agreed that LCoGS indicators as initially listed were too vague to allow for comparability across or within multiple settings, and their data elements had never been defined. However, we were unanimous that the indicators themselves were useful, especially when used together as a set to assess timely access to quality surgical, obstetric, and anaesthesia care. We also agreed that data points should evolve over time and account for the development of countries’ ability to collect data, or the different uses of data, recognising the trade-offs between the need for granular data collection and the difficulties of collecting these more granular data, including the resource limitations to enable this collection. This “evolution” also enables different uses of those data, with, at current time, the basic data points—which should be collectable by most countries—used for advocacy, including international or national comparisons, and the intermediate or full data points being of greater utility for national planning in countries that have the current resources to collect them or for research studies [5,13]. Given the broader utility of indicators derived from and disaggregated according to the full data points, we urge researchers working at local, national, or regional levels to use these definitions in order to later allow compilation of data from across multiple countries using systematic methodologies and meta-analyses. Additionally, although we recognise that these more granular data points (of intermediate and full) may not be feasible to collect in countries where data systems are nascent, we strongly recommend they be collected for national planning purposes as soon as possible.

To achieve political priority for anaesthesia and surgery, and, hence, funding for global surgery development and research requires that 4 elements are in place in the broad areas of (i) actor power; (ii) ideas; (iii) political context; and (iv) issue characteristics (broadly, the capture of data to show the issues that need to be addressed) [31–33]. The global surgery movement has been shown to be deficient in all of these areas, especially in comparison to the movement to improve maternal health [31]. This Utstein meeting was convened to address, in particular, the area of issue characteristics, while also including global surgery actors to ensure the findings of the meeting and resultant indicators—when collected—are used to improve the provision of surgical care globally. The use of a basic set of harmonised data will facilitate coherent presentation of ideas and their internal and external framing and enable a shift in the political context to provide the funding for more detailed data collection for the intermediate and full set of indicators.

### Strengths and limitations of the process

Our process has many strengths. The Utstein consensus process is well established for the development of guidelines; the consensus process was rigorous, with each indicator discussed by 2 small working groups, then agreed among a larger working group. The indicators went on to be discussed in plenary and further refined after the meeting if needed. Experts in clinical practice, academia, and the use of metrics for policy making or global reporting were represented to ensure that metrics are both collectable and utilisable at national and international levels for change in practice and global advocacy.

However, there were limitations of our approach. Importantly, representatives from LMICs were limited (with 8 out of the 38 actively working in LMICs). This was due to a combination of the limited space, our need to include people working in global health metrics (the World Bank, WHO, UN, and IHME)—most of whom are based in high-income countries, and difficulties in travel for some others; nevertheless, most attendees had experience of living and working in LMICs. Additionally, those actively working in LMICs at the time of the meeting were respected Global Surgery leaders with influence beyond the facility or country in which they work. Lastly, that discussions after the meeting in Utstein had to be held virtually, rather than in person, could be considered a limitation. However, these meetings were greatly facilitated by the growth in adoption of online meeting platforms.

## Conclusions

Access to surgical and anaesthetic care is crucial for ensuring the health and wealth of populations. Global reporting of accessible, comparable, and utilisable data is central to ensuring advocacy, and the newly defined indicators will facilitate such data collection. These refinements may also improve collection of more granular data to inform national policy making. These updated indicator definitions applied at the international and national level will facilitate progress towards advocating for and achieving timely access to safe, affordable care, and for setting evidence-based targets. An eventual output of this process should be directly actionable by individual countries and the United Nations Statistical Commission, with broad and long-term international impact. We have started the next phase of the process—to work with partners in LMICs to ascertain how to collect the evolved indicators in in-country settings and the digital infrastructure required for this data collection.

## Supporting information

S1 File **Table A**. The original Lancet Commission on Global Surgery (LCoGS) indicators. **Table B**. Working group leads and Steering Committee of the Utstein meeting. **Text A**. Points of Disagreement. **Tables C-G**. Basic, intermediate, and full sets of data points for each indicator as well as current data sources.
(DOCX)

## Abbreviations

ASA: American Society of Anaesthesia
LCoGS: The Lancet Commission on Global Surgery
LMIC: low- or middle-income country
POMR: perioperative mortality rate
SAO: surgical, anaesthesia, and obstetric

## References

[1] Meara JG, Leather AJ, Hagander L, Alkire BC, Alonso N, Ameh EA, et al. Global Surgery 2030: evidence and solutions for achieving health, welfare, and economic development. Lancet. 2015;386(9993):569–624. doi: 10.1016/S0140-6736(15)60160-X 25924834

[2] Mock CN, Donkor P, Gawande A, Jamison DT, Kruk ME, Debas HT, et al. Essential surgery: key messages from Disease Control Priorities, 3rd edition. Lancet. 2015;385(9983):2209–19. doi: 10.1016/S0140-6736(15)60091-5 25662414 PMC7004823

[3] WHO. Global Reference List of 100 Core Health Indicators (plus health-related SDGs). Geneva: WHO; 2018.

[4] The World Bank. World Development Indicators 2019. Available from: http://data.worldbank.org.

[5] Hanna JS, Herrera-Almario GE, Pinilla-Roncancio M, Tulloch D, Valencia SA, Sabatino ME, et al. Use of the six core surgical indicators from the Lancet Commission on Global Surgery in Colombia: a situational analysis. Lancet Glob Health. 2020;8(5):E699–710. doi: 10.1016/S2214-109X(20)30090-5 32353317

[6] Nataraja RM, Yin Mar O, Andolfatto L, Moore EM, Watters D, Aye A, et al. Analysis of Financial Risk Protection Indicators in Myanmar for Paediatric Surgery. World J Surg. 2020;44(12):3986–92. doi: 10.1007/s00268-020-05775-w 32920705

[7] Guest GD, McLeod E, Perry WRG, Tangi V, Pedro J, Ponifasio P, et al. Collecting data for global surgical indicators: a collaborative approach in the Pacific Region. BMJ Glob Health. 2017;2(4):e000376. doi: 10.1136/bmjgh-2017-000376 29225948 PMC5717952

[8] Watters DA, Guest GD, Tangi V, Shrime MG, Meara JG. Global Surgery System Strengthening: It Is All About the Right Metrics. Anesth Analg. 2018;126(4):1329–39. doi: 10.1213/ANE.0000000000002771 29547428

[9] Tran TM, Fuller AT, Butler EK, Makumbi F, Luboga S, Muhumuza C, et al. Burden of Surgical Conditions in Uganda: A Cross-sectional Nationwide Household Survey. Ann Surg. 2017;266(2):389–99. doi: 10.1097/SLA.0000000000001970 27611619

[10] Biccard BM, Madiba TE, Kluyts HL, Munlemvo DM, Madzimbamuto FD, Basenero A, et al. Perioperative patient outcomes in the African Surgical Outcomes Study: a 7-day prospective observational cohort study. Lancet. 2018;391(10130):1589–98. doi: 10.1016/S0140-6736(18)30001-1 29306587

[11] Holmer H, Bekele A, Hagander L, Harrison EM, Kamali P, Ng-Kamstra JS, et al. Evaluating the collection, comparability and findings of six global surgery indicators. Br J Surg. 2019;106(2):e138–e50. doi: 10.1002/bjs.11061 30570764 PMC6790969

[12] Juran S, Gruendl M, Marks IH, Broer PN, Guzman JM, Davies J, et al. The need to collect, aggregate, and analyze global anesthesia and surgery data. Can J Anaesth. 2019;66(2):218–29. doi: 10.1007/s12630-018-1261-5 30484168

[13] Massenburg BB, Saluja S, Jenny HE, Raykar NP, Ng-Kamstra J, Guilloux AGA, et al. Assessing the Brazilian surgical system with six surgical indicators: a descriptive and modelling study. BMJ Glob Health. 2017;2(2):e000226. doi: 10.1136/bmjgh-2016-000226 28589025 PMC5444087

[14] Peberdy MA, Cretikos M, Abella BS, DeVita M, Goldhill D, Kloeck W, et al. Recommended guidelines for monitoring, reporting, and conducting research on medical emergency team, outreach, and rapid response systems: an Utstein-style scientific statement: a scientific statement from the International Liaison Committee on Resuscitation (American Heart Association, Australian Resuscitation Council, European Resuscitation Council, Heart and Stroke Foundation of Canada, InterAmerican Heart Foundation, Resuscitation Council of Southern Africa, and the New Zealand Resuscitation Council); the American Heart Association Emergency Cardiovascular Care Committee; the Council on Cardiopulmonary, Perioperative, and Critical Care; and the Interdisciplinary Working Group on Quality of Care and Outcomes Research. Circulation. 2007;116(21):2481–500. doi: 10.1161/CIRCULATIONAHA.107.186227 17993478

[15] Idris AH, Berg RA, Bierens J, Bossaert L, Branche CM, Gabrielli A, et al. Recommended guidelines for uniform reporting of data from drowning: the "Utstein style". Circulation. 2003;108(20):2565–74. doi: 10.1161/01.CIR.0000099581.70012.68 14623794

[16] Idris AH, Bierens J, Perkins GD, Wenzel V, Nadkarni V, Morley P, et al. 2015 Revised Utstein-Style Recommended Guidelines for Uniform Reporting of Data From Drowning-Related Resuscitation: An ILCOR Advisory Statement. Circ Cardiovasc Qual Outcomes. 2017;10(7). 10.1161/HCQ.0000000000000024 PMC6168199 28716971

[17] Jacobs I, Nadkarni V, Bahr J, Berg RA, Billi JE, Bossaert L, et al. Cardiac arrest and cardiopulmonary resuscitation outcome reports: update and simplification of the Utstein templates for resuscitation registries: a statement for healthcare professionals from a task force of the International Liaison Committee on Resuscitation (American Heart Association, European Resuscitation Council, Australian Resuscitation Council, New Zealand Resuscitation Council, Heart and Stroke Foundation of Canada, InterAmerican Heart Foundation, Resuscitation Councils of Southern Africa). Circulation. 2004;110(21):3385–97. doi: 10.1161/01.CIR.0000147236.85306.15 15557386

[18] Moher D, Schulz KF, Simera I, Altman DG. Guidance for developers of health research reporting guidelines. PLoS Med. 2010;7(2):e1000217. doi: 10.1371/journal.pmed.1000217 20169112 PMC2821895

[19] Chao TE, Sharma K, Mandigo M, Hagander L, Resch SC, Weiser TG, et al. Cost-effectiveness of surgery and its policy implications for global health: a systematic review and analysis. Lancet Glob Health. 2014;2(6):e334–45. doi: 10.1016/S2214-109X(14)70213-X 25103302

[20] Weiser TG, Gawande A. Excess Surgical Mortality: Strategies for Improving Quality of Care. In: Debas HT, Donkor P, Gawande A, Jamison DT, Kruk ME, Mock CN, editors. Essential Surgery: Disease Control Priorities. 3rd ed (1). Washington (DC); 2015. doi: 10.1596/978-1-4648-0346-8_ch16 26740999

[21] Weiser TG, Haynes AB, Molina G, Lipsitz SR, Esquivel MM, Uribe-Leitz T, et al. Size and distribution of the global volume of surgery in 2012. Bull World Health Organ. 2016;94(3):201–9F. doi: 10.2471/BLT.15.159293 26966331 PMC4773932

[22] Weiser TG, Makary MA, Haynes AB, Dziekan G, Berry WR, Gawande AA, et al. Standardised metrics for global surgical surveillance. Lancet. 2009;374(9695):1113–7. doi: 10.1016/S0140-6736(09)61161-2 19782877

[23] Kruk ME, Gage AD, Arsenault C, Jordan K, Leslie HH, Roder-DeWan S, et al. High-quality health systems in the Sustainable Development Goals era: time for a revolution. Lancet Glob Health. 2018;6(11):E1196–E252. doi: 10.1016/S2214-109X(18)30386-3 30196093 PMC7734391

[24] Davies JI, Meara JG. Global surgery—going beyond the Lancet Commission. Lancet. 2015;386(9993):507–9. doi: 10.1016/S0140-6736(15)60465-2 25924835

[25] Davies JI, Vreede E, Onajin-Obembe B, Morriss WW. What is the minimum number of specialist anaesthetists needed in low-income and middle-income countries? BMJ Glob Health. 2018;3(6):e001005. doi: 10.1136/bmjgh-2018-001005 30588342 PMC6278919

[26] Stones W, Visser GHA, Theron G, Motherhood FS, Newborn HC. FIGO Statement: Staffing requirements for delivery care, with special reference to low- and middle-income countries. Int J Gynaecol Obstet. 2019;146(1):3–7. doi: 10.1002/ijgo.12815 30927443

[27] WHO U, UNFPA, AMDD. Monitoring emergency obstetric care, a handbook. WHO: WHO; 2009.

[28] Odland ML, Nepogodiev D, Morton D, Martin J, Bekele A, Ghosh D, et al. Identifying a Basket of Surgical Procedures to Standardize Global Surgical Metrics: An International Delphi Study. Ann Surg. 2020. doi: 10.1097/SLA.0000000000004611 33214454

[29] Ariyaratnam R, Palmqvist CL, Hider P, et al. Toward a standard approach to measurement and reporting of perioperative mortality rate as a global indicator for surgery. Surgery. 2015;158:17–26. doi: 10.1016/j.surg.2015.03.024 25958067

[30] Palmqvist CL, Ariyaratnam R, Watters DA, et al. Monitoring and evaluating surgical care: defining perioperative mortality rate and standardising data collection. Lancet. 2015 Apr 27;385 Suppl 2:S27. doi: 10.1016/S0140-6736(15)60822-4 Epub 2015 Apr 26. 26313074

[31] Shawar YR, Shiffman J, Spiegel DA. Generation of political priority for global surgery: a qualitative policy analysis. Lancet Glob Health. 2015;3(8):e487–e95. doi: 10.1016/S2214-109X(15)00098-4 26187491

[32] Smith SL, Shiffman J. Setting the global health agenda: The influence of advocates and ideas on political priority for maternal and newborn survival. Soc Sci Med. 2016;166:86–93. doi: 10.1016/j.socscimed.2016.08.013 27543685 PMC5034850

[33] Shiffman J, Shawar YR. Strengthening accountability of the global health metrics enterprise. Lancet. 2020;395(10234):1452–6. doi: 10.1016/S0140-6736(20)30416-5 32305072 PMC7162633

