# Supplementary material for: Global surgery, obstetric, and anaesthesia indicator definitions and reporting: An Utstein consensus report
Source: PLoS Med. 2021 Aug 20;18(8):e1003749. doi: 10.1371/journal.pmed.1003749 (PMC8415575; doi:10.1371/journal.pmed.1003749)
Supplement: S1 File — Table A. The original Lancet Commission on Global Surgery (LCoGS) indicators. Table B. Working group leads and Steering Committee of the Utstein meeting. Text A. Points of Disagreement. Tables C-G. Basic, intermediate, and full sets of data points for each indicator as well as current data sources. (DOCX) [file pmed.1003749.s001.docx]

**Supporting information**

**Table A: The original Lancet Commission on Global Surgery (LCoGS) indicators.**

| Indicator | Definition | Target |
| --- | --- | --- |
| Group 1: Preparedness for surgery and anaesthesia care | | |
| 1. Access to timely essential surgery | Proportion of the population that can access, within 2 hours, a facility that can do caesarean delivery, laparotomy, and treatment of open fracture (the Bellwether Procedures) | A minimum of 80% coverage of essential surgical and anaesthesia services per country by 2030 |
| 2. Specialist surgical workforce density  (WDI indicator [SH.MED.SAOP.P5](https://data.worldbank.org/indicator/SH.MED.SAOP.P5)) | Number of specialist surgical, anaesthetic, and obstetric physicians who are working, per 100 000 population | 100% of countries with at least 20 surgical, anaesthetic and obstetric physicians per 100 000 population by 2030 |
| Group 2: delivery of surgical and anaesthesia care | | |
| 3. Surgical volume  (WDI Indicator [SH.SGR.PROC.P5](https://data.worldbank.org/indicator/SH.SGR.PROC.P5)) | Surgical procedures^a^ done in an operating theatre, per 100 000 population per year | 80% of countries by 2020 and 100% of countries by 2030 tracking surgical volume; a minimum of 5000 surgical procedures per 100 000 population by 2030 |
| 4.Perioperative mortality | All-cause death rate before discharge in patients who have undergone a surgical procedure^a^ in an operating theatre, divided by the total number of surgical procedures, presented as a percentage | 80% of countries by 2020 and 100% of countries by 2030 tracking perioperative mortality; in 2020, assess global data and set national targets for 2030 |
| Group 3: Effect of Surgery and Anaesthesia | | |
| 5. Protection against impoverishing expenditure (WDI Indicator [SH.SGR.IRSK.ZS](https://data.worldbank.org/indicator/SH.SGR.IRSK.ZS)) | Proportion of households protected against impoverishment^b^ from direct out-of-pocket payments for surgical and anaesthesia care | 100% protection against impoverishment from out-of-pocket payments for surgical and anaesthesia care by 2030 |
| 6. Protection against catastrophic expenditure (WDI Indicator [SH.SGR.CRSK.ZS](https://data.worldbank.org/indicator/SH.SGR.CRSK.ZS)) | Proportion of households protected against catastrophic expenditure^c^ from direct out-of-pocket payments for surgical and anaesthesia care | 100% protection against catastrophic expenditure from out-of-pocket payments for surgical and anaesthesia care by 2030 |
| ^a^ A surgical procedure is defined as the incision, excision, or manipulation of tissue that needs regional or general anaesthesia, or profound sedation to control pain. This may include inpatient, outpatient, and ambulatory procedures.  ^b^ using a threshold of $1.25 purchasing power parity/day  ^c^ 10 percent of total income | | |

**Table B: Working group leads and Steering Committee of the Utstein meeting**

| Tom Weiser Access Working Group Chair |
| --- |
| Justine Davies Access Working Group Co-Chair and Steering Committee |
| Hampus Holmer Workforce Working Group Chair |
| Julian Gore-Booth Workforce Working Group Co-Chair and Steering Committee |
| David Watters Surgical Volume Working Group Chair |
| John Meara Surgical Volume Working Group Co-Chair and Steering Committee |
| Janet Martin POMR Working Group Chair and Steering Committee |
| Bruce Biccard POMR Working Group Co-Chair |
| Emmanuel Makasa Expenditure Working Group Chair |
| Mark Shrime Expenditure Working Group Co-Chair |
| Andy Leather Parking Lot Working Group Chair |
| Adrian Gelb Parking Lot Working Group Co-Chair and Steering Committee |
| Jannicke Mellin-Olsen Steering Committee |

**Text A: Points of Disagreement.**

There were four points of disagreement which required discussion in small – virtual - working groups.

1. POMR

One panel member expressed concern that requiring facilities to report POMR would result in pejorative judgements and punitive actions towards individuals working in that facility. Their preference was that POMR is reported globally as a dichotomous variable, “collected or not”, in any country, rather than the POMR outcomes collected. Whilst others felt that POMR is important to report as it is the only clinical outcome among the Global Surgery indicators. Collection of POMR is now widely accepted in high income country settings where it is used in morbidity and mortality meetings as well as to inform service delivery at a national level. Its collection for research has shown vast inequalities in access to quality surgery care across settings, which are likely to drive advocacy efforts to increase funding for provision of quality surgery. After discussion, the group agreed that the indicator should be reported globally, at the country level and not at the individual facility level.

POMR was also discussed in relation to the lack of risk adjustment in the basic set of indicators – which makes comparison between settings challenging. However, the group agreed that although non-adjusted results should be considered with caution, they were still valuable to collect to produce comparisons over time for individual countries, give an indication of comparisons between settings, and initiate the process of collecting and reporting this important indicator.

1. Use of Bellwethers

The group agreed that the Bellwethers were not well defined and there was ongoing discussion about whether to use these throughout the definitions in the indicators or to develop a list of procedures to inform a basic “basket” of procedures – akin to that which is used to calculate the consumer price index. We agreed after small group discussion to tighten up the definition of the Bellwethers and that they should remain included as they are accepted widely by the Global Surgery Community. However, to consider evolving this to a basket of procedures, once these have been developed and gained acceptance by the Global Surgery Community. Work has now been done to develop these via an international Delphi process and their use in the LCoGS metrics will be discussed at future meetings.

1. Definitions of workforce

The group agreed that defining and capturing practitioners of surgery and anaesthesia care is essential, but there are multiple definitions and that these are challenging to capture and harmonise. Whilst there was no disagreement as such about these definitions, discussions to develop definitions which were clear and reflected cadres that work in multiple countries were iterative. The definitions presented in this manuscript are those which were agreed upon by all meeting attendees.

1. Timeframe for evolution of the indicators

The timeframe for the evolved metrics (intermediate and full) was also discussed, with some arguing that 2 and 5 years was not feasible in many low resourced settings. However, we agreed that these should be included to act as a stimulus to countries that are starting to collect indicators.

**Tables C-G Basic, Intermediate, and Full sets of data points for each indicator as well as current data sources.**

Table C: Geospatial access

| **Indicator 1: Geospatial Access** | | | |
| --- | --- | --- | --- |
| **LCoGS indicator definition** | Proportion of the population that can access, within 2 hours, a facility that can do caesarean delivery, laparotomy, and treatment of open fracture (the Bellwether procedures) | | |
| **Utstein revised definition** | **Proportion of a country’s population with geographic access (within 2 hours) to a facility capable of providing surgical and anaesthesia care for the Bellwether procedures (caesarean section, laparotomy, and surgical management of open long bone fracture)** | | |
| **Overall summary of data elements** | - **Population estimates** - **Facility locations** - **Capacity of health facilities to do Bellwether procedures** - **Distance and travel time of population to facilities** | | |
|  | **Basic** | **Intermediate** | **Full** |
| **Data points needed to construct the indicator** | Population  - Population data or modelled estimates at resolution of 1x1km (by 5 year age groupings and sex, if available)  Facility location/capability  - Location of health facilities offering Bellwether procedures  Distance/travel time(3.1)  - Estimated time to travel to facilities from population locations | Population  - Population data or modelled estimates at resolution of 1x1km, by 5 year age groupings and sex  Facility location  - Location of all health facilities  Surgical capability  - Health facility capacity to perform LCoGS Bellwethers based on facility reports, preferably validated by site assessment. Facilities to be classed as capable if doing at least one of each Bellwether procedure in the last month  Distance/travel time(3.1)  - Estimated time to travel to facilities from population locations  Facility type  - Public or private  - Primary, secondary, or tertiary | Population  - Population data or modelled estimates at resolution of 1x1km, by 5 year age groupings and sex  Facility location  - Location of all health facilities  Surgical capability  - Health facility capacity to perform LCoGS Bellwethers based on facility reports validated by site assessment. Facilities to be classed as capable if doing at least one of each LCoGS Bellwether procedure in the last month  - Number and type of each surgical procedure done (with harmonised surgical procedure code)  Distance/travel time  - Verified user-generated travel time and source of transport (3.3)  Facility type  - Public or private  - Primary, secondary, or tertiary  Realised access  - Household data regarding need for and access of surgical services (3.4) |

The Basic data sets are for use for global reporting at the macro-level only since they provide insufficient granularity to inform national planning or service refinement at the meso- or micro-level.

(3.1) Note, for comparability, travel time means ideal time to travel between a location and a facility, not experienced travel time from recognition of the need for surgery to arriving at a facility, which may incorporate delays in seeking care or delays in obtaining transport.

For comparability across countries we also recommend that Open Street Maps (OSM) is used, accepting that reliability of results from OSM may differ by country (results may be more reliable in countries where transport time is less affected by weather, for example). However, for national use, verified travel times are likely to be preferable.

(3.2) Data on procedures are widely captured in paper form even in the most under-resourced settings; these data are not widely available electronically. To be usable, these need to be electronically maintained and standards for reporting (including procedure codes) harmonised using agreed standard procedure codes. We envision that will occur in the Full data set (>5 year timeframe), with regular reporting to Ministry of Health (MoH)

(3.3) Verified actual travel times collected by local survey are not currently widely collected outside of the research setting; these could be collected as part of census data

(3.4) Data for realised access is not yet available outside of research studies. It could be possible to capture this via population census data e.g. on experienced travel times, modes of transport, and need for and access to surgery care. Need for surgery care will likely be self-reported (“have you been told that you needed an operation?”), given logistical difficulties in verifying surgical need

(3.5) Data on facility type are not widely available, do exist in some countries, for others, this variable can be introduced to current collection tools

Table D: Workforce

**Indicator 2: Workforce**

| **LCoGS indicator definition** | Number of specialist surgical, obstetric, and anaesthetic physicians who are working per 100 000 population. | | |
| --- | --- | --- | --- |
| **Utstein revised definition** | **Number of each of surgery, obstetric, or anaesthesia providers who are actively practicing, per 100 000 population** | | |
| **Overall summary of data elements** | - **Provider(4.1) numbers as:** - Number of *nationally certified(4.2) specialist-physician(4.3) practitioners* of each cadre of surgery, obstetric, or anaesthesia care, excluding trainees - Number of *nationally* c*ertified non-specialist physician practitioners* of surgery, obstetric, or anaesthesia care, excluding trainees - Number of *nationally certified non-physician practitioners* of surgery, obstetric, or anaesthesia care, excluding trainees - Number of *other practitioners (“other practitioners”)* of surgery, obstetric, or anaesthesia care who do not fit into aforementioned categories (includes physician trainees and non-certified non-physician providers) - **Total country population** | | |
|  | **Basic** | **Intermediate** | **Full** |
| **Data points needed to construct the indicator** | Providers  - Total number of nationally certified specialist physician practitioners for each of surgery, obstetric or anaesthesia care  Disaggregated by cadre (surgery, obstetric or anaesthesia providers)  - Total number of other nationally certified providers of surgery, obstetric, or anaesthesia care (not nationally certified physician practitioners)  Disaggregated by cadre (surgery, obstetric or anaesthesia providers)  Population  - Total country population | Providers(4.4)  - Total number of nationally certified specialist physician, non-specialist physician, non-physician, or “other practitioners” of surgery, obstetric or anaesthesia care  Disaggregated by:  -Certified providers, disaggregated by:  specialist physician, non-specialist physician practitioners, or non-physician practitioners  - Certified providers, disaggregated by cadre (surgery, obstetric, or anaesthesia providers)  - Nationally certified non-physician practitioners, disaggregated by type of provider (nurse, medical officer etc.)  - Geographical location of practitioners practice (at least for the macroscopic level of rural or urban)  - Employment in public or private practice  - In a defined level of facility (tertiary, secondary, primary care)  Population  - Geographical location of population within the country (at least for the macroscopic level of rural or urban)(4.5) | Providers(4.4)  - Total number of nationally certified specialist physician, non-specialist physician, non-physician, or “other practitioners” of surgery, obstetric or anaesthesia care  Disaggregated by:  -Certified providers, disaggregated by:  specialist physician, non-specialist physician, or non-physician practitioners  - Certified providers, disaggregated by cadre (surgery, obstetric, or anaesthesia providers)  - Nationally certified and other non-certified, practitioners, disaggregated by type of provider (nurse, medical officer, etc)  - Other practitioners, disaggregated by cadre (surgery, obstetric, or anaesthesia care)  - Geographical location of practitioners practice (at least for the macroscopic level of rural or urban)  - Employment in public or private practice  - In a defined level of facility (tertiary, secondary, primary care)  - Age of providers  - Sub-specialisation of providers  Population  Geographical location of population within the country (at least for the macroscopic level of rural or urban)(4.5) |

The Basic data sets are for use for global reporting at the macro-level only since they provide insufficient granularity to inform national planning or service refinement at the meso- or micro-level.

(4.1) We have not provided a definition of what a surgery, anaesthetic, or obstetric provider is; we agreed these should be defined by each country, recognising that the definitions are likely to vary locally. Providers are persons directly involved in delivering the surgical, obstetric, or anaesthetic care; i.e. the person doing the operation or giving the anaesthetic.

(4.2) Certified means completion of a government and/or professionally approved advanced education program that leads to a nationally recognised qualification to provide surgery, anaesthesia, or obstetric care.

(4.3) Specialist physicians are providers who have obtained a medical degree (physician) and undergone specialty post-graduate training (certification)

(4.4) The terminology used to describe providers is not currently consistent across data sources. We estimate standardisation will be possible after the 2 year timeframe for the Intermediate and Full data set. The data sources available are not consistently granular enough to allow the above provider-type disaggregated variables to be captured. However, could readily be adjusted to include more granular variables

(4.5) Geographical location of providers and population will enable assessment of whether the distribution of the population is matched by that of the providers

Table E: Volume

**Indicator 3: Volume**

| **LCoGS indicator definition** | Number of procedures done in an operating theatre, per 100 000 population per year | | |
| --- | --- | --- | --- |
| **Utstein revised definition** | **Number of surgical procedures done in an operating theatre using any form of anaesthesia(5.1), per 100,000 population per year** | | |
| **Overall summary of data elements** | - **Number of surgical procedures done in an operating theatre, using any anaesthesia, per year** - **Total country population** | | |
|  | **Basic** | **Intermediate** | **Full** |
| **Data points needed to construct the indicator** | Surgical procedures  - Total number of surgical procedures done in an operating theatre using any form of anaesthesia(5.1) per year  Population  - Total country population | Surgical procedures  - Total number of surgical procedures done in an operating theatre using any form of anaesthesia(5.1) per year  Disaggregated by:  - Age and sex of patient  - ASA class  - Emergency or elective  - Type of surgical procedure(5.2)  - Rural or urban location  - Facility type (Public or private, or primary, secondary, or tertiary)  Population  - Total country population  - Population living in the catchment area(5.3) of each identified hospital | Surgical procedures  - Total number of surgical procedures done in an operating theatre using any form of anaesthesia(5.1) per year  Disaggregated by:  -Age and sex of patient  - ASA class  - Emergency or elective  - Type of surgical procedure and surgical procedure code(5.2)  - Diagnosis(5.4)  - Rural or urban location  - Facility type (Public or private, or primary, secondary, or tertiary)  - Number of reoperations (5.5)  - Level of certification of practitioner providing the surgical, anaesthetic or obstetric care (see workforce)  Population  - Total country population  - Population living in the catchment area(5.3) of each identified hospital |

The Basic data sets are for use for global reporting at the macro-level only since they provide insufficient granularity to inform national planning or service refinement at the meso- or micro-level. ASA refers to American Society of Anaesthesiology.

(5.1) This recognises that at current time, definitions of procedures that constitute surgery differ among countries and data sources. We have therefore agreed upon a broad definition for surgical procedures (without defining a list)

This definition includes incision, excision, or manipulation of tissue using anaesthesia in an operating theatre, including day-cases but excluding surgical procedures in other locations i.e. outside of the operating room

Definition of anaesthesia is regional or general anaesthesia, or profound sedation to control pain during the procedure

If surgical procedures are coded (for example, using International Classification of Diseases [ICD]), any number of surgical codes used during a single anaesthetic is counted as one case

(5.2) Data on procedures are widely captured in paper form even in the most under-resourced settings; these data are not widely available electronically. To be useable, these need to be electronically maintained and standards for reporting (including procedure codes) harmonised using agreed standard procedure codes. We envision that will occur in the Full data set (>5 year timeframe), with regular reporting to MoH

For caesarean sections, the total number of vaginal and caesarean deliveries, at the country level for the Basic data set, and in the population living in the catchment area of a facility (see below for definition of catchment area) in the Intermediate and Full data sets is needed to enable calculation of a caesarean section rate (denominator being number of deliveries)

(5.3) *Catchment area* refers to the population who would normally attend the facility in question. This should be locally defined

(5.4) Diagnosis is often captured, but we recommend using a standardised, harmonised system, for example, ICD 11

(5.5) Data on whether emergency/elective surgical procedure, requirement for reoperation, and level of certification of provider providing care are not consistently collected in log books currently. However, existing tools (logbooks, records, and operating theatre registries) can be readily adjusted to capture this information

Table F: Perioperative Mortality Rate

**Indicator 4: Perioperative Mortality Rate (POMR)**

| **LCoGS indicator definition** | All-cause death rate before discharge in patients who have undergone a surgical procedure in an operating theatre using any form of anaesthesia, divided by the total number of surgical procedures, presented as a percentage, per year | | |
| --- | --- | --- | --- |
| **Utstein revised definition** | **Deaths from all-causes, before discharge (up to 30 days), in all patients who have received any anaesthesia for a surgical procedure done in an operating theatre(6.1), divided by the total number of procedures, per year, expressed as a percentage.** | | |
| **Overall summary of data elements** | - **Number of patients undergoing a surgical procedure using any form of anaesthesia who died before hospital discharge, per year** - **Number of surgical procedures done in an operating theatre, using any anaesthesia, per year (from Indicator 3, Volume)** | | |
|  | **Basic** | **Intermediate** | **Full** |
| **Data points needed to construct the indicator** | Deaths  - Total number of deaths in all patients who received any anaesthesia for a surgical or obstetric procedure,(6.1) per year  Surgical procedures(6.2)   - - Total number of surgical procedures done using any anaesthesia per year   Time point:   - - Deaths before discharge | Deaths  - Total number of deaths in all patients who received any anaesthesia for a surgical or obstetric procedure,(6.1) per year  Surgical procedures(6.2)   - - Total number of surgical procedures done using any anaesthesia per year   Time point:   - - Deaths before discharge   Disaggregated by: (6.4)  - Age and sex of patient  - ASA class  - Emergency or elective  - Type of surgical procedure(6.2)  - Rural or urban location  - Facility type (Public or private, or primary, secondary, or tertiary) | Deaths  - Total number of deaths in all patients who received any anaesthesia for a surgical or obstetric procedure,(6.1) per year  Surgical procedures(6.2)   - - Total number of surgical procedures done using any anaesthesia per year   Time point:   - - Deaths before discharge - - Deaths within 30 days after the procedure date(6.5)   Disaggregated by:(6.4)  -Age and sex of patient  - ASA class  - Emergency or elective  - Type of procedure and procedure code(6.2)  - Diagnosis (6.3)  -Rural or urban location(6.6)  - Facility type (Public or private, or primary, secondary, or tertiary)  - Number of reoperations  - Level of certification of practitioner providing the surgical, anaesthetic or obstetric care (see workforce) |

The Basic data sets are for use for global reporting at the macro (or country) -level only since they provide insufficient granularity to inform national planning or service refinement at the meso- or micro-level. For example, the Basic data set does not provide meaningful comparison of POMR across settings since the results are not adjusted for baseline patient risk or type of procedure.

(6.1) This recognises that at current time, definitions of procedures that constitute surgery differ between countries and data sources. We have therefore agreed upon a broad definition of procedures for the Basic data set (<2 year time frame), without defining a list.

This definition includes incision, excision, or manipulation of tissue under any form of anaesthesia in an operating theatre. This includes day-cases, but excludes procedures in other locations i.e. outside of the operating theatre

Definition of anaesthesia is regional or general anaesthesia, or profound sedation to control pain

Number of surgical codes in a single anaesthesia procedure counted as one case.

If only a subset of procedures is feasible for this indicator, then the type of procedures included should be transparently reported.

(6.2) Data on procedures are widely captured in paper form even in the most under-resourced settings; these data are not widely available electronically. To be useable, these need to be electronically maintained and standards for reporting (including procedure codes) harmonised using agreed standard procedure codes. We envision that will occur in the Full data set (>5 year timeframe), with regular reporting to MoH

(6.3) Diagnosis is often captured, but we recommend using a standardised, harmonised system, for example, ICD 11

(6.4) Data on whether emergency/elective procedure, requirement for reoperation, and level of certification of provider providing care are not consistently collected. However, existing tools (logbooks, records, and OR registries), can be adjusted to capture this information

(6.5) For 30-day follow-up, where this is not routinely reported, discrete studies including periodic prospective sampling may be necessary to collect representative data that could be modelled to project country-wide data

(6.6) knowledge of distance of patients’ residence to facility also has utility in determining whether there was a potential delay in accessing surgery that contributed to the individual’s death. This is not currently collected or available, but could feasibly be calculated from patient’s addresses on medical records

Table G: Financial Risk Protection

**Indicator 5: Financial Risk Protection (FRP)**

| **LCoGS indicator definition** | Financial Risk Protection: “Risk of Catastrophic Expenditure from Surgical Care” | | |
| --- | --- | --- | --- |
| **Utstein revised definition** | **Percentage of the population at risk of catastrophic expenditure *if* they were to require surgical care(7.1)** | | |
| **Overall summary of data elements** | - ***Out of pocket expenditure (OOP)(7.2)***   OOP is the *direct medical* costs incurred from receiving surgical care from time of admission to a facility capable of providing surgical and anaesthesia care to the time of discharge.   - **Household expenditure**   Total household expenditure (Y) is defined as “the sum of the monetary values of all items (goods and services) consumed by each household” over 12 months.   - **Catastrophic expenditure threshold**   The catastrophic expenditure threshold should be set at 10% of total household expenditure.(7.3)  ***If (OOP/Y)x100 >10, catastrophic expenditure has occurred*** | | |
| **Time frame and importance** | **Basic** | **Intermediate** | **Full** |
| **Data points needed to construct the indicator** | OOP expenditure for access to surgical care   - Nationally-representative survey of direct OOP expenditure   Household expenditure   - National total household expenditure (per individual household) | OOP expenditure for access to surgical care   - Nationally-representative survey of direct OOP expenditure   Household expenditure   - National total household expenditure (per individual household) | OOP expenditure for access to surgical care  Nationally-representative survey of direct OOP expenditure   - Disaggregated by procedure - Individual-level OOP   Additionally:   - Total health expenditure (THE) for the care episode including pre-hospital direct medical costs, direct non-medical costs (lodging, food, transport) and indirect costs (loss of earnings, loss of crops) - - Funding for healthcare (health insurance contributions)   Household expenditure   - National total household expenditure (per individual household) - District level total household expenditure (per individual household) |

The Basic data sets are for use for global reporting at the macro-level only since they provide insufficient granularity to inform national planning or service refinement at the meso- or micro-level.

(7.1) Catastrophic expenditure is usually calculated at the individual level (with data collected on OOP and household expenditure for each individual undergoing a medical admission episode). However, many people do not access surgery care because of fear of catastrophic expenditure. This indicator thus uses individual OOP expenditure in combination with national average level household expenditure to estimate the proportion of people who would suffer catastrophic expenditure *if* they were to need surgery

(7.2) Direct OOP costs, in reality, could include pre-hospital direct medical costs. However, they are not included here as they are small relative to the hospitalisation episode and patients may not recall these as readily as hospitalisation costs.

OOP should not be derived from hospital billing alone as this will substantially under-estimate OOP

This does not include direct non-medical costs (lodging, food, transport to and from facility). This does not include indirect costs (e.g.: loss of earnings)

(7.3) We note as per SDG Target 3.8.2 there are two recognised thresholds, >10% and > 25%, however, we have chosen 10%.
